# Carboplatin-based Nanomedicine to Enhance the Anticancer Effect in SK-NEP-1 WilmsꞌTumor Cells

**Published:** 2017

**Authors:** Ming Ming, Zhao-long Ma, Yong-tao Xu, Feng-yin Sun, Xin-hai Cui

**Affiliations:** a *Department of Pediatric Surgery, Qilu Hospital, Shandong University, 107#, Wenhua Xi Road, Jinan, 250000 P.R. China. *; b *Department of Pediatric Surgery, Taian City Central Hospital, Taian, 271000, P.R. China. *

**Keywords:** Carboplatin, Apoptosis, Cell proliferation, Wilm’s tumor, Anticancer effect

## Abstract

Wilms tumor (WT) is the most common pediatric malignant primary renal tumor. Carboplatin (CRB), a platinum compound is widely used in the treatment of multiple cancers including ovarian, lung, head and neck, and wilm’s tumor. However, lower uptake of CRB in cancer cells and toxicity concerns in healthy cells often limited its clinical outcome. The aim of this study was to investigate the antitumor effect of CRB on SK-NEP-1 wilm’s cancer cells. Earlier, CRB was formulated in nanoparticulate formulations and characterized its biophysical parameters. SK-NEP-1 cell growth in vitro was assessed by MTT. Then, apoptosis potential was investigated by TUNEL, Hoechst, and colony formation assay. CRB treatment resulted in inhibition of cell proliferation of SK-NEP-1cells in a dose-dependent manner. TUNEL, Hoechst, and colony formation assay demonstrated that CRB was more effective in killing wilm’s cancer cell when encapsulated in nanoparticle formulations. Overall, the present study demonstrates that CRB treatment resulted in marked inhibition of cell proliferation and cell apoptosis. These results may pave way for the effective treatment of wilm’s tumor in clinical models.

## Introduction

Wilm’s tumor is one of most common form of primary malignancies in kidney of pediatric patients (1). Wilms tumor specifically affects children in the first 2-3 years of their life. The present day advancement in understanding of molecular basis of this tumor has increased the overall survival rate to more than 85% with good clinical outcomes (2-4). A better knowledge of stimuli and signaling pathways involved with this tumor progression could be a great boon in the treatment of Wilms tumor (5, 6). Although this renal cancer is curable with a multiple combination of surgery, chemotherapy, and radiotherapy, it resulted in severe complications in pediatric patients and some cases children poor responded to this kind of treatment (7, 8). Therefore, there is a sincere need to develop good therapeutic strategies to increase the tumor treating efficiency while reducing the associated complications. 

In this regard, carboplatin (CRB), a platinum compound is widely used in the treatment of multiple cancers including ovarian, lung, head and neck, and wilm’s tumor (9, 10). The CRB is often preferred over cisplatin due to the lower incidence of nephrotoxicity and ototoxicity (11). Carboplatin is a second generation analog of cisplatin, with which it shares various structural and pharmacological characteristics. CRB exhibits its therapeutic effect via induction of apoptosis and cancer cell death (12). However, lower uptake of CRB in cancer cells and toxicity concerns in healthy cells often limited its clinical outcome (13). Nanotechnology-based solutions open new window in molecular oncology which assures to change the fate of preexisting drugs in the intracellular environment (14). The nanoparticles with altered biodistribution and improved pharmacokinetics are reported to reduce the side effects and to increase the therapeutic efficacy by accumulating in a higher concentration in the tumor cells by hiding the drug from plasma proteins or macrophages to ensure longevity in body fluids (15). Chitosan nanoparticles are one such delivery system which is expected to increase the cellular uptake of CRB and so is the therapeutic efficacy (16) Chitosan is an excellent polymer which is highly biodegradable and biocompatible and proven delivery vehicle in cancer targeting.

Therefore, in the present study, we have designed novel carboplatin-loaded chitosan nanoparticles (CRB NP) to increase the cancer treatment efficiency in wilm’s tumor. The main aim of the present study was to increase the cellular uptake of CRB in renal cancer cells while reducing its side effects. The antitumor efficacy of free CRB and CRB NP was evaluated by means of various biological assays including cytotoxicity assay, cell invasion assay, Tunnel based apoptosis assay, and Hoechst-staining based apoptosis assay.

## Experimental

*Morphological imaging*

The CRB-loaded chitosan nanoparticles were prepared as reported previously. The morphological examination of CRB NP was carried out through a high resolution transmission electron microscopy (TEM, Hitachi^®^ 800 MT, and Japan). The liquid samples were counterstained with phosphotungistic acid and placed over a carbon-coated copper grid and air-dried. The particles were further investigated by means of atomic force microscopy (AFM).

*Cell culture conditions*

SK-NEP-1 Human kidney (Wilm´s Tumor) cell line obtained from the American Type Culture Collection (ATCC) was maintained in the Maccyo’5 (Life Technologies Inc., Gaithersburg, MD, USA) supplemented with 20% heat-inactivated fetal bovine serum (Invitrogen Co., NY, USA) in a humidified incubator with 5% CO2 at 37 °C.

*Cytotoxicity assay*

The cytotoxicity potential of CRB and CRB NP was evaluated by means of MTT (3-(4, 5-dimethylthiazol-2-yl)-2, 5-diphenyltetrazolium bromide) (MTT assay). 1×10^4^ cells were seeded in a 96-well plate and incubated overnight. Next day, cells were treated with experimental formulations and further incubated for 24 h. 20uL MTT solution (5 mg/mL) was added to each well and incubated at 37 °C for a further 4 h. Then 200 uL of DMSO was added to each well after the medium was removed. The optical density (OD) values were measured at 490 nm on a scanning multi-well spectrophotometer (BioRad Model 550 USA).

*Colony forming assay*

5×10^5^ cells were seeded in a 96-well plate and incubated overnight. Next day, the cells were treated with experimental formulations and further incubated for 24 h. The colonies were then allowed to grow for 14 days. without any disturbance. The colonies were stained with 1% methylene blue and then counted. The experiments were performed in triplicate, and each experiment was reproduced.

*Apoptosis assay- Hoechst staining and TUNEL assay*

The apoptosis effect of formulations on the cancer cell was investigated by means of Hoechst staining as well as TUNEL assay. Briefly, 5×10^5^ cells were seeded in a 96-well plate and incubated overnight. Next day, cells were treated with experimental formulations and further incubated for 24 h For Hoechst assay, treated cells were stained with Hoechst 33342 for 10 min at 23 °C. The amount of apoptosis cells were then investigated by means of fluorescence microscope. The strong fluorescence and condensed or fragmented nuclei were observed in the nuclei of apoptotic cells, whereas weak fluorescence was observed in live cells. Quantification of apoptotic cells was performed by taking the images in random fields and counting at least 300 cells in four random fields in each well.

For TUNEL assay, incubated cells were treated with experimental formulations and further incubated for 24 h Then, the next day, they were incubated with TUNEL solution and 4′, 6-diamidino-2-phenylindole (DAPI) solution at 37 °C for 60 min without light. The cells were then observed under fluorescence microscope. Green fluorescence was observed in the nuclei of apoptotic cells. All cells showed blue fluorescence for DAPI.

*Statistical analysis*

Statistical analysis was performed using GraphPad Prism (version 5.01, GraphPad Software, Inc., CA, USA) statistical software. The Student’s t-test was used to analyze significance between independent groups. The significance was accepted as *P* < 0.05.

## Results and Discussion

*Evaluation of carboplatin therapeutic delivery system*

**Figure 1:**
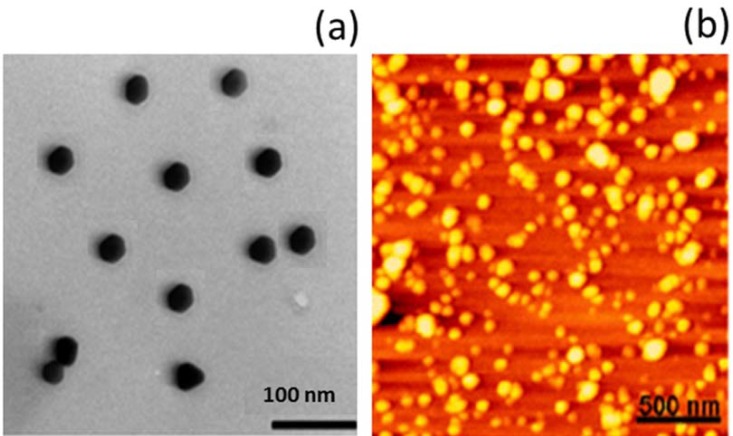
Morphology analysis of CRB nanoparticles; (A) transmission electron microscopy (TEM) images; (B) atomic force microscopy (AFM) images

Wilms tumor specifically affects children in the first 2-3 y. of their life. Although this renal cancer is chemotherapy and radiotherapy curable with a multiple combination of surgery, it resulted in severe complications in pediatric patients and in some cases, the children responded to this kind of treatment. In this perspective, carboplatin (CRB) a platinum compound is widely used in the treatment of multiple cancers including ovarian, lung, head and neck as well as wilm’s tumor. CRB exhibits its therapeutic effect via induction of apoptosis and cancer cell death. However, lower uptake of CRB in cancer cells and toxicity concerns in healthy cells often limited its clinical outcome. In the present study therefore, we have developed a unique therapeutic carrier which is capable of increasing the therapeutic efficacy of the drug while it can reduce the associated side effects. Chitosan nanoparticle has been proven to be very effective in cancer treatment; however, its efficacy is none tested in wilm’s tumor. Therefore, we dela with CRB NP and have characterized its physicochemical properties. At first, the particle size of CRB NP was around 160 nm which is very much suitable for cancer targeting. A particle size less than 200 nm is required for the efficient cancer targeting. In this study, TEM analysis was performed to observe its morphological appearances (Figure 1A). As seen, particles were clearly spherical shaped with clear boundaries with each other. Such a spherical particle is expected to increase the *in-vivo* performance. The shape was further confirmed by the AFM imaging wherein particles were circular and seen flattened on the mica surface (Figure 1B). From the morphological analysis, it is confirmed that a circular to spherical shaped particle is formed and the drug is incorporated in the core of the nanoparticles.

**Figure 2:**
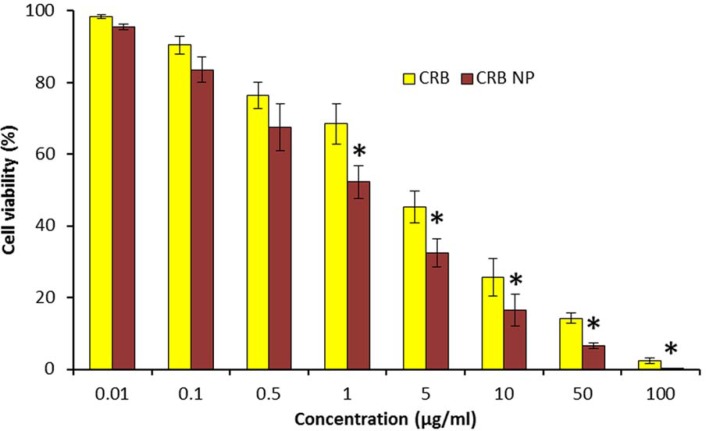
*In-vitro* antitumor efficacy of free CRB and CRB NP in wilm’s tumor cells. The cytotoxicity effect of CRB was investigated by means of MTT assay and presented as dose vs % cell viability

**Figure 3:**
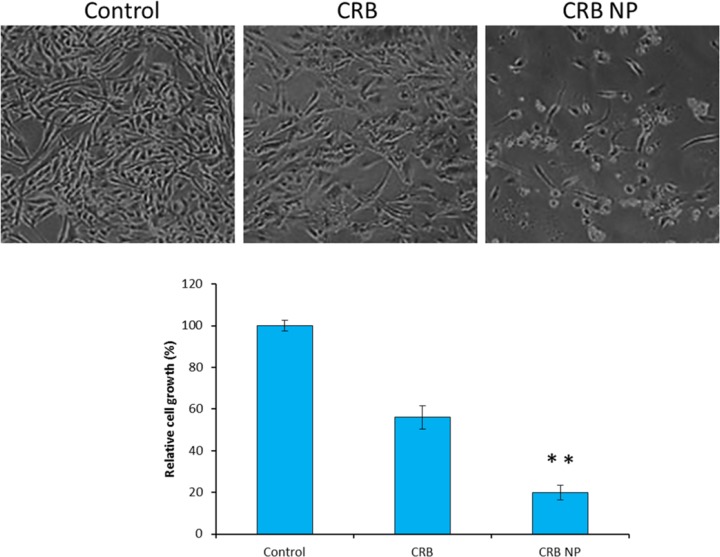
Morphological images of cells after treatment with specific formulations. The cells were observed under optical microscope and followed by cells were counted by trypan blue dye

**Figure 4:**
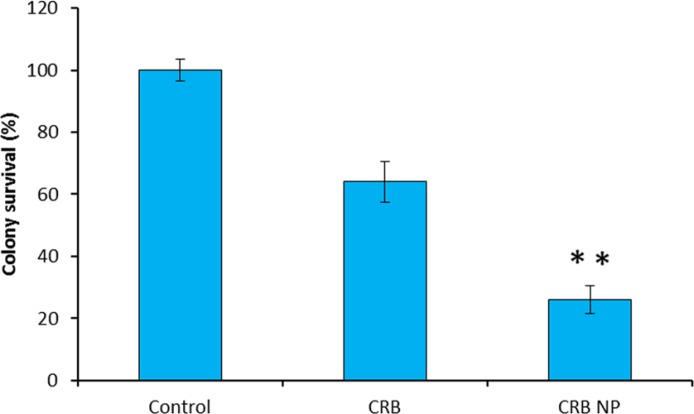
Colony formation assay. The anticancer effect of different formulations on the colony formation ability was tested in cancer cells. The cells were grown in the absence of drug, and allowed to form colonies for 14 days

**Figure 5:**
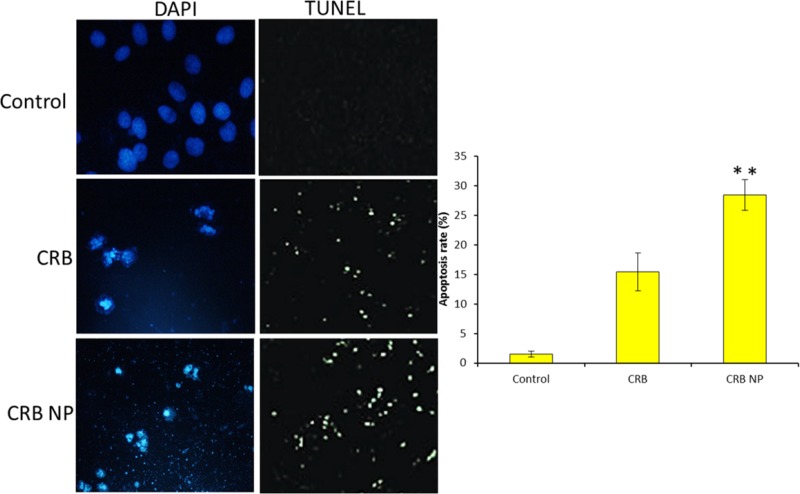
*In-vitro* antitumor efficacy of free CRB and CRB NP was evaluated by means of (a) Hoeschst staining and (b) TUNEL assay. The blue fluorescence in the cells indicates the Hoeschst staining and for TUNEL assay, green fluorescence was observed from the nucleus

*Cytotoxicity potential of carboplatin in SK-NEP-1 Wilm’s tumor cell*

The cytotoxic effect of CRB and CRB-based nanosystem was evaluated by means of MTT assay (Figure 2). The CRB formulations showed a strong dose-dependent cytotoxic effect in SK-NEP-1 wilms cancer cells. It has to be noted that the cytotoxic effect of CRB NP was far greater than that of free CRB in the cancer cells. For example, at 1 µg/mL of drug dosage, the cell viability of CRB and CRB NP were remained at 72% and 56%, respectively. Similar results were seen at 10 µg/mL where in cell viability of CRB and CRB NP were remained at 34% and 18%, respectively. The strong anticancer effect of CRB NP was associated with its ability to internalize more efficiently in the cancer cells whereas free CRB might not penetrate effectively as evident from the cytotoxic data. This suggests that incorporation of CRB in NPs potentiates the cytotoxic propensity of CRB and also 2 times reduction in the concentration to bring 50% cytotoxicity was obtained. Overall, a strong anticancer effect of CRB NP in the *in-vitro* level could guarantee its 

Efficacy in the *in-vivo* level.

*Morphological changes in the SK-NEP-1 cell*

The intracellular morphological changes were observed by optical microscopy (Figure3). For this, cells were treated with respective formulations and incubated for 24 h The cells were then washed and observed under optical microscope. One can see that untreated control cells maintained their typical morphology and spread over the entire cover slip. More cells were uniformly spread without any sign of apoptosis. In contrast, appreciable changes in cell morphology were observed in case of CRB treated cells. The cells were less in number and also irregular indicating the typical anticancer effect of free CRB on the cancer cells. Most tellingly, CRB NP showed a superior antitumor effect in this cancer cell with a greater apoptosis of cancer cells. It can be clearly seen that more than 80% of cells were unadhered and the remaining cells were round in shape which is a typical sign of cell apoptosis. The results were very much consistent with the cytotoxicity assay results. Trypan blue dye exclusion assay was further done to count the number of cells remaining which was then correlated with the number of cells in control and then cell growth was estimated. As seen, CRB NP showed 80% reduction in the cell growth while CRB reduced like 40% of cell growth. These results further portray the superior anticancer effect of nanoparticle loaded drug than the free drugs. 

*Colony formation assay*

Tumor progression occurs through invasion of tumor cells into surrounding normal tissue. Migratory ability of tumor cells is also responsible for metastasis, a key process that aids in tumor propagation (Figure 4). The colony formation assay was performed to investigate the ability of CRB and CRB NP to inhibit the colony formation. The control group which does not have the drug showed the 100% colony formation without any inhibition. On the contrary, cells treated with CRB and the encapsulated one significantly inhibited the colony formation ability. Mainly, one can see that CRB NP was more efficient in having ability to form colony in the *in-vitro* conditions. Specifically, CRB NP showed 70% ability to inhibit the colony formation while CRB showed only 35% ability after 14 days. of incubation.

*Apoptosis assay*

To confirm the apoptosis potential of different formulations, TUNEL assay and Hoechst based apoptosis were evaluated. It can be clearly seen that cells treated with CRB NP showed more apoptosis features compared to that of control. The Hoechst staining assay showed that the fluorescent intensity of control cells was highly fluorescent whereas CRB and CRB NP treated cells showed marked decrease of the fluorescence intensity indicating the apoptosis of cancer cells (Figure 5a). Furthermore, TUNEL assay also showed the similar results consistent with the Hoechst based apoptosis analysis. The quantitative data further showed that CRB NP exhibited nearly 30% of cancer cell apoptosis whereas CRB showed only 15% of apoptosis and control showed 2% of cancer cell apoptosis (Figure 5b). The results from both studies suggest that CRB has promising antitumor activity against SK-NEP-1 cells. This work indicated that CRB is an important targeting agent for human Wilms tumor cells. 

## Conclusions

In conclusion, we have demonstrated the anticancer effect of CRB and CRB-loaded nanoparticles on SK-NEP-1 wilm’s tumor cell. The therapeutic delivery system was observed to be nanosized and shown to be suitable for the cancer therapeutics. Our cytotoxicity data revealed a tremendous anticancer effect of CRB in cancer cells justifying its potential role in the treatment of wilms tumor. Colony formation assay showed the superior inhibitory of CRB NP in the cancer cells while TUNEL assay showed a superior anticancer effect of nanoparticles with marked cell death pattern. Overall, our study provided new clues for the treatment of wilm’s tumor in human.
